# Das Zahnærztliche Institut Der Kœnig-Lichen Universitæt Zu Berlin

**Published:** 1885-09

**Authors:** W. D. Miller


### DAS ZAHNÆRZTLICHE INSTITUT DER KŒNIG-LICHEN UNIVERSITÆT ZU BERLIN.

BY PROFESSOR W. D. MILLER.

I have been repeatedly asked by persons studying dentistry in America, and purposing to take the degree of D. D. S. there, what the requisites are for admission to the State Dental Examination in Berlin, and in what branches the candidates for the title "Approbirter Zahnarzt" are examined. In order to furnish information on this point to all inquirers at once, as well as to explain briefly the manner in which the Institute is conducted, this article is written.

The Berlin Dental Institute is a part of the University, and all students of dentistry at the Institute must be matriculated there. As ladies may not matriculate at the University, they cannot be admitted to the study of dentistry at the Institute. Foreigners are matriculated on presenting a passport alone, without any special reference to their previous education. Persons of German nationality, however, are required to furnish evidence of having passed the examinations for the Prima of a German Gymnasium, or first class Realschule, which is equivalent to a preparation for the Freshman year at the best American Colleges and Universities, and if required at our American Dental Schools would exclude a very large proportion of the applicants for admission. Foreigners wishing to attempt the State Dental Examination are required to present a petition to the Kultus-Minister, who determines the conditions for admission to an examination in such cases. Candidates of German nationality must furnish, in addition to the requisites for admission given above, evidence of having had four semesters of University study, and practical exercise in operative and mechanical dentistry. Four semesters (18 months) are equivalent to four years in the majority of American Dental Schools.

It will be seen that very few of that class of Germans who flock to America to pursue their studies in dentistry can expect to be admitted to examinations here; first, because they do not possess the necessary general education, and second, because they have not taken the prescribed four semesters. The possession of a D. D. S. will not be of any assistance whatever, since this title does not have any more value here than any other two letters of the alphabet put together in any order whatever. On the contrary, a German possessing the degree of D. D. S., who is not an "Approbirter Zahnarzt," is in danger of being looked upon as one who, not having the ability or energy to make his examination here, has escaped to America, where it is believed that every one may go through with the greatest ease, and in the shortest possible time.

The term Dentist, or Dentiste, applied to the possessor of a D. D. S., is little less than a term of reproach, and such a dentist who calls himself "Zahnarzt" is liable to prosecution. A few days since, four persons, possessing diplomas from American dental colleges, were prosecuted and fined for putting the term "Amerik Zahnarzt" on their signs. The Deans of the dental colleges do well in taking steps to remedy the evils for which they themselves are measurably responsible, but they must take many and long strides before they succeed in removing from American Dentistry the stigma which some of them have helped to bring upon it. There are schools in America which still command respect here, as well as at home ; they are too well known to need particular mention.

The Dental Institute, which was opened only last October, and which was a new experiment in Germany, has thus far proved a success. The rooms temporarily occupied have become over-filled, and new ones are already in requisition. For the summer semester over eighty students of dentistry matriculated at the University. A new building, devoted exclusively to dental purposes, is promised, but we shall probably be obliged to wait a few years before we see its completion. Lectures on anatomy, physiology, surgery, materia medica, etc., are heard at the University, while the work at the Institute is carried on in three separate departments. The first, conducted by Prof. Busch, comprises oral surgery, including extraction; the second, conducted by Professors Miller and Pætsch, comprises the conservative treatment of the diseases of the teeth and their nearer complications; the third, mechanical dentistry, is conducted by Prof. Sauer.

In all these departments the work is done under the eye of the professor and assistant, both spending at least two hours daily in the clinic rooms. In this respect the students of dentistry here are more favored than in America, where the acting professor may not be seen in the operating room more than two or three times during the whole year. The instruction given in the operating rooms is essentially the same as that in American schools, except that in some respects it is more general, in that it endeavors to give practice in all the methods of using gold, both cohesive and non-cohesive—even Herbst's method not being left out in the cold altogether—while no method or preparation of gold is recommended to the exclusion of all others. Tin and gold combined, as well as the plastics, amalgam, etc., all very useful in their places, are brought into requisition.

There is a marked difference between the entering class in the Berlin school and that in an American school. The former have a better general education, and are better prepared to enter upon the study of any subject of a scientific nature, while the latter are much farther advanced in practical dentistry, and many of them, who are sons of dentists, or have worked for months or years in dental offices, are able to make very good gold operations on entering. Here, however, to be the son of a Zahnarzt, or to have been in practice for a number of years, does not by any means necessarily signify the ability to insert a gold filling, to properly prepare a cavity, or to adjust the cofferdam. "Approbirter Zahnarzts" come to us to learn those things which many American students know and do on entering college; nevertheless, Zahnarzt is a title of which every possessor is proud, because it represents a certain number of years devoted to general education, and a number of semesters devoted to the special study of dentistry, while D. D. S. is altogether an uncertain quantity, and may represent either capacity or incapacity, learning or ignorance, as the case may be.

In conclusion, no one who cannot fulfill the requirements stated in the beginning of this article need make application for admission to examination, as the laws are very stringent, and exceptions are virtually never made, and, as a warning to those who disappear from amongst us for a short time, and, returning a few weeks later, bring back with them laurel wreaths from certain American colleges that, in Germany, have gained more of notoriety than reputation, we will say that both they and the institutions that crowned them are in continual danger of being exposed. Unfortunately, as is too often the case, the innocent must suffer with the guilty, and even those properly graduated from schools in good standing are not always exempt.

